# Seroprevalence of *Leishmania* spp. in Cattle Breeds of the Mediterranean Region: Effect of the Breed in the Immune Response

**DOI:** 10.1155/tbed/3277232

**Published:** 2025-03-05

**Authors:** Lola Martínez-Sáez, Vicenzo Lopreiato, Luigi Liotta, Carmelo Cavallo, Annalisa Amato, Pablo Jesús Marín-García, Lola Llobat

**Affiliations:** ^1^Molecular Mechanisms of Zoonotic Diseases (MMOPS) Research Group, Department of Animal Production and Health, Public Health and Food Science and Technology (PASAPTA), Medicine Veterinary Faculty, Universidad Cardenal Herrera-CEU, CEU Universities, Valencia, Spain; ^2^Department of Veterinary Sciences, University of Messina, Messina, Italy; ^3^Department of Animal Production and Health, Public Health and Food Science and Technology (PASAPTA), Medicine Veterinary Faculty, Universidad Cardenal Herrera-CEU, CEU Universities, Valencia, Spain

**Keywords:** bovine breed, cattle, cytokine, immune response, *Leishmania*

## Abstract

*Leishmania* spp. is an intracellular obligate protozoan that causes the zoonotic disease leishmaniosis. Although the dog has always been considered the main reservoir, the number of species involved in transmission of the parasite is increasingly numerous and includes both domestic species, such as cats or horses, wildlife species, and livestock such as pigs, sheep, or cows. In the latter, the presence of *Leishmania* spp. has been detected in some countries of South America, Asia, and Africa. In Europe, and specifically in the Mediterranean region where leishmaniasis is endemic, there are no data in this regard, although cow blood has been detected in sandflies, which act as the vector for this parasite. This study analyzed the seroprevalence of *Leishmania* spp. in 75 lactating cows of three different cattle breeds (Modicana, Simmental, and Holstein) from Southern Italy, finding an overall seroprevalence of 17.33%. Cytokine serum levels related to immune response were analyzed and the presence of *Leishmania* spp. infection did not change the levels of cytokines interleukin 1*β* (IL-1*β*), IL-6, IL-10, interferon gamma (IFN-*γ*), and tumor necrosis factor alpha (TNF-*α*). Interaction between breed and infection was observed, the IL-1*β* being higher in Modicana breed than in Simmental and Holstein when infection was present. This breed had medium levels of IL-6 without infection, with high levels being observed in Simmental and low levels in Holstein. Furthermore, Simmental cows showed higher levels of IL-6 with infection than without infection. These results suggest that the livestock species could play a relevant role in *Leishmania* spp. transmission in endemic regions, and with different immune responses depending on the breed. Additional research is required to ascertain the role of livestock species in parasite transmission and evaluate the immune response of autochthonous breeds.

## 1. Introduction

Leishmaniasis, caused by the protozoan *Leishmania* spp., indeed poses a significant public health concern in many parts of the world. The parasite is transmitted by the bites of infected sandflies, which serve as vectors. Different species of *Leishmania* spp. have been adapted to different sandfly species in various regions, with different species acting as vectors in the Old and New Worlds, and can be anthroponotic or zoonotic depending on the region [1]. The life cycle of *Leishmania* spp. is indeed complex, involving two primary stages, the promastigote stage within the invertebrate vector (sandfly) and the amastigote stage within the vertebrate host (either human or animal) [2]. This adaptability and complexity contribute to the challenges in controlling and treating the disease effectively. Moreover, the transmission dynamics of leishmaniasis can vary, with transmission routes classified as anthroponotic (primarily involving humans) or zoonotic (involving animals). Understanding these transmission dynamics is crucial for implementing effective control measures tailored to the specific epidemiological situation in each region. Research and surveillance efforts play a vital role in monitoring vector populations, identifying new vector species, and developing strategies for disease control and prevention. This multifaceted approach is essential to mitigate the burden of leishmaniasis on public health worldwide [3].

The wide range of hosts that can harbor and potentially transmit *Leishmania* spp. parasites adds another layer of complexity to leishmaniasis epidemiology. At least 70 species of wild and domestic animals have been identified as confirmed or potential reservoirs of the *Leishmania* parasite worldwide [4]. These reservoir hosts play a critical role in the maintenance and transmission of the parasite within endemic regions. Wild animals, as well as domestic animals like dogs and cats or livestock, can serve as reservoirs for various *Leishmania* spp., depending on the geographical area [5]. Identifying these reservoir hosts is essential for understanding the transmission dynamics of leishmaniasis and implementing effective control measures.

The role of livestock species, and specifically the role of cattle as reservoir of *Leishmania* spp., has been studied in recent years in endemic regions, and different species of *Leishmania* have been found in cattle breeds in different parts of the world, including Brazil [6], Iran [7], India [8], Nepal [9], Bangladesh [10], Ethiopia [11], Sudan [12], and Thailand [13]. In the Mediterranean region, where leishmaniasis is endemic, no studies on prevalence of *Leishmania* spp. infection have been reported, although cattle blood has been found in the phlebotomine sandfly in Tunisia [14] and Italy [15, 16].

The aim of this study is to investigate the seroprevalence of *Leishmania* spp. in different cattle breeds in Sicily and determine the effect of the breed on seroprevalence and cytokine serum levels.

## 2. Material and Methods

### 2.1. Animals and Sample Collection

A total of 74 animals living in Sicily (Southern Italy, Mediterranean region) were included in this study. None of animals presented any clinical signs and were apparently healthy. Cows included in the study were of three different breeds: 20 Holstein (dairy cattle breed), 30 Simmental (dual-purpose cattle breed), and 24 Modicana (not specialized autochthonous cattle breed of the Sicilian region) lactating cows. All animals living in a commercial dairy farm under the traditional semi-intensive farming system.

Samples were obtained by venipuncture of the jugular vein and collected into 10 mL tubes containing clot activator and separating gel (Terumo Corporation, Tokyo, Japan). Blood samples were centrifuged for 10 min at 2000 × *g* and the supernatant serum was collected and stored at −20°C until further analysis.

### 2.2. *Leishmania infantum* Analysis

Serum samples were used to test for the presence of specific *Leishmania* spp. antibodies for *Leishmania* spp. in each sample. Following the manufacturer's instructions, specific antibodies were found using the *Leishmania* vet ELISA test for anti-*Leishmania* specific immunoglobulin G (IgG) antibodies (Demeditec Diagnostic GmbH, Bonn, Germany). An animal was deemed seropositive if its ELISA titer was greater than the cut-off. For every test, there were both positive and negative controls. The kit's sensitivity and specificity are >98%, according to the manufacturer.

### 2.3. Cytokine Serum Levels Measure

Serum levels of different cytokines were measured. Interleukin (IL)-1*β*, IL-6, IL-10, interferon gamma (IFN-*γ*) and tumor necrosis factor alpha (TNF-*α*) (Cattle IL-1*β* ELISA kit, Cattle IL-6 ELISA kit, Cattle IL-10 ELISA kit, Cattle IFN-*γ* ELISA kit, and Cattle TNF-*α* ELISA kit, MyBioSource, San Diego, USA) levels were measured in serum samples by commercial ELISA kits method following the manufacturer's recommendations. Briefly, a 100 µL serum sample was used for the analysis with sandwich-ELISA. An antibody that was specific to cytokines was used to precoat the microplate. The microplate wells were filled with the samples and the corresponding antibody. Next, each microplate well was filled with an avidin-horseradish peroxidase (HRP) conjugate and a biotinylated detection antibody specific for each cytokine. The microplate wells were then incubated. Free parts were removed by washing. To every well, substrate solution was added. Using the Victor-X3TM plate reader (Perkin Elmer), the optical density (OD) and spectrophotometric measurements of the enzyme–substrate reaction were made at a wavelength of 450 nm. By comparing the sample OD to the standard curve, the concentration of each cytokine was determined.

### 2.4. Statistical Analysis

Pearson's Chi-square test was carried out to analyze the prevalence of *Leishmania* spp. infection. After normality and homoscedasticity checking with Shapiro–Wilks and Levène tests, respectively, ANOVA test was performed to determine the association of *Leishmania* spp. infection and breed, and to correlate infection with cytokine serum levels. The correlation between cytokine serum levels was analyzed by Pearson's test correlation. The statistical program SAS (North Carolina State University, Cary, CA, USA) was used for the statistical analysis and differences were considered statistically significant at *p*-value < 0.05.

## 3. Results

**Figure 1:**
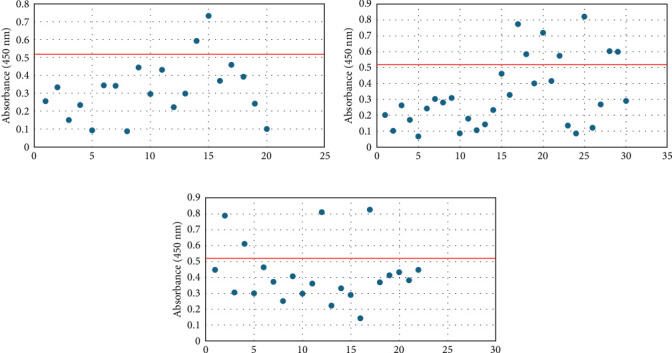
Scatter diagrams for the absorbance values at 450 nm obtained for each animal according to its breed in the ELISA technique for the detection of *Leishmania* spp. and the cut-off of the technique (0.519 nm): (a) Holstein cows; (b) Simmental cows; and (c) Modicana cows.

**Figure 2:**
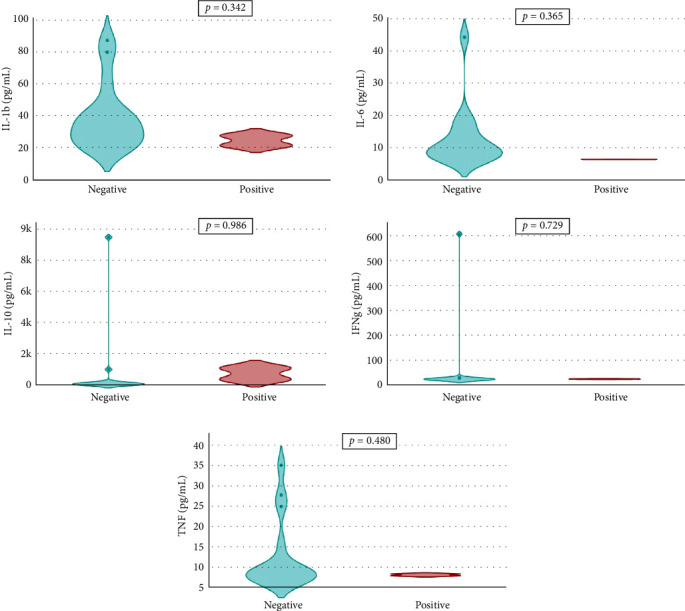
Serum levels of interleukin 1*β* (IL-1b), IL-6, and IL-10. Interferon-*γ* (IFNg) and tumor necrosis factor-α (TNF) in seronegative and seropositive Holstein cows.

**Figure 3:**
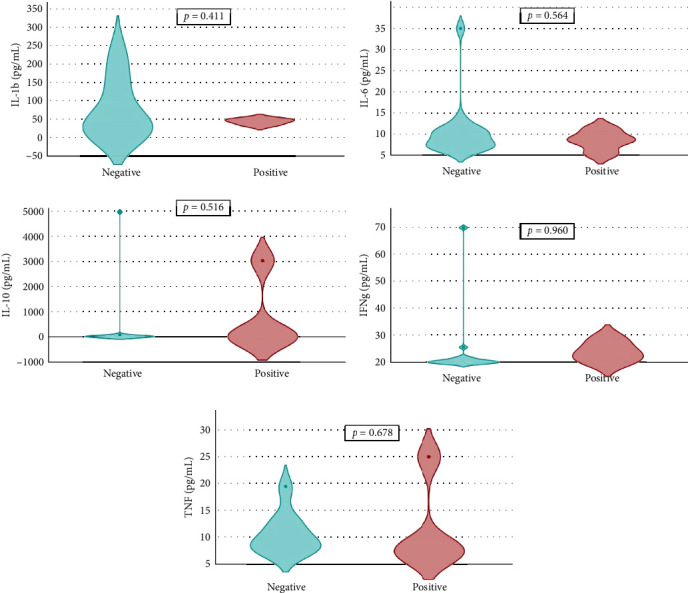
Serum levels of interleukin-1*β* (IL-1b), IL-6, and IL-10. Interferon-*γ* (IFNg) and tumor necrosis factor-*α* (TNF) in seronegative and seropositive Modicana cows.

**Figure 4:**
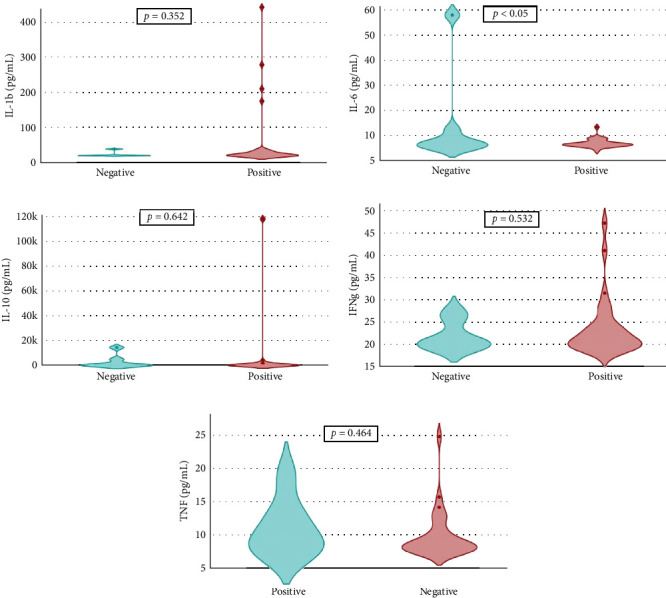
Serum levels of interleukin-1*β* (IL-1b), IL-6, and IL-10. Interferon-*γ* (IFNg) and tumor necrosis factor-*α* (TNF) in seronegative and seropositive Simmental cows.

Overall seroprevalence was 17.33% in animals included, and no breed effect was found (Table 1). The scatter diagrams of the absorbance values for each of the animals according to their breed, as well as the cut-off of the ELISA technique, are shown in Figure 1. Cytokine serum levels were similar between seropositive (29.93 ± 1.22 pg/mL, 11.43 ± 14.09 pg/mL, 1866.96 ± 4064.52 pg/mL, 22.58 ± 3.16 pg/mL, and 10.83 ± 5.41 pg/mL for IL-1*β*, IL-6, IL-10, IFN-*γ*, and TNF-*α*, respectively) and seronegative animals (58.67 ± 78.56 pg/mL, 9.38 ± 6.35 pg/mL, 4232.73 ± 20996.01 pg/mL, 32.52 ± 74.12 pg/mL, and 10.83 ± 5.44 pg/mL for IL-1*β*, IL-6, IL-10, IFN-*γ*, and TNF-*α*, respectively) (Table S1). Elevated dispersion in IL-10 serum levels was found in all measures. An interaction between bovine breed and infection was found. In fact, the Modicana breed had higher levels of IL-1*β* than Holstein or Simmental in seropositive animals (Table 2), whereas in seronegative, Simmental presented higher levels of IL-6 than Modicana, and Modicana showed higher levels than Holstein (Table 3). Within each breed analyzed, no differences in cytokine serum levels were found between seropositive and seronegative animals in Holstein (Figure 2) and Modicana (Figure 3) cows. In Simmental breed, seropositive animals showed lower levels of IL-6 than seronegative cows (Figure 4).

Pearson correlation coefficients show in Table 4 (seropositive animals) and Table 5 (seronegative animals). No statistical correlation between cytokine serum levels was found. Changes in the sign of the correlation were found between some cytokines depending on infection or noninfection. High levels of IL-1*β* were correlated to high levels of IFN-*γ* and low levels of IL-6 and TNF-*α*, but only in seropositive animals. In seronegative animals, high levels of IL-1*β* were correlated to high levels of IL-6 and TNF-*α*. Within of each breed analyzed, only a statistical positive correlation was found between IL-1*β* and IL-6 serum levels in Modicana cows, with a correlation coefficient of 0.51 (*p* < 0.05).

## 4. Discussion

In this study, seroprevalence of *Leishmania* spp. infection was analyzed in cattle for the first time in the Mediterranean basin. A possible limitation of the study is that the tests were performed with an ELISA test that is not specific to the bovine species. However, some studies show that the detection of antibodies against *Leishmania* spp. with ELISA tests is reliable, even in other species as cats [17], wild rabbits [18] or wild animals as guarani (*Speothos venaticus*), wild canids (*Chrysocyon* brachyurus, *Cerdocyon thous*, Pseudalopex vetulus), and raccoon [19]. Results showed a total seroprevalence of 17.33%, without differences between the three breeds studied. Molecular detection of *Leishmania* spp. in cattle has been reported in some countries such as India [8], Nepal [9], and Ethiopia [11], where the prevalence was estimated at around 1%. However, a higher seroprevalence was found in other African countries, reaching up to 21.4% in Sudan [12]. Although there are no seroprevalence data for *Leishmania* spp. in cows in the Mediterranean region, different studies have shown the presence of blood from this species in the parasite's transmitting vector. In fact, Remadi et al. [14] studied the feeding habits of sandflies in Tunisia and showed that the majority of sandflies were found to have fed on cattle (39.05%), followed by humans. In Italy, low percentages were found, around 4.7% [16] in the northern and around 2.3% in the southern region [15]. In view of these results, the role of cattle as a reservoir of *Leishmania* spp. should be considered, since there is only one report of clinical signs associated with infection by this parasite in cows. This clinical case, reported in Switzerland, reported cutaneous leishmaniosis, characterized by ulcers and nodules, deep cutaneous mycosis, cutaneous tumors, and erythema multiforme [20]. These clinical signs can often go unnoticed in cattle, so they could be acting as a silent reservoir.

Control of *Leishmania* spp. infection in mammals has been related to a protective immune response mediated by Th1 cells. Th1 cells play a crucial role in activating macrophages, which are important immune cells that can kill pathogens like parasites [21]. The main cytokines involved in this pathway are TNF-*α*, IFN-*γ*, IL-1*β*, IL-2, and IL-6 [22, 23]. The pathway mediated by Th2 lymphocytes can be activated and trigger the humoral response, with stimulation of cytokines IL-4, IL-5, and IL-10, generating an excess of antibodies [24, 25]. In our study, *Leishmania* spp. seropositivity did not seem to modify the serum levels of the cytokines evaluated in cows, which could indicate an immune system prepared to respond quickly to infection by the parasite in this species. High dispersion was found in IL-10 serum levels in all animals included, suggesting an activation of the Th2 response in some animals.

Differences in immune response are related to cattle breed, so Modicana animals infected showed higher levels of IL-*β* than other breeds. This cytokine has a protective effect against *Leishmania* spp. infection, so this breed native to Southern Sicily presented a more protective immune response against infection. Similar results have been found in Rendena cattle, an autochthonous Italian breed whose high expression levels of IL-1*β* in epithelial cells and leukocytes are related to mastitis resistance [26]. When the infection is not present, Simmental and Modicana cows have higher levels of IL-6 than Holstein, which could indicate an immune system more adapted to parasite infection in these two breeds. Genes related to parasitic infection resistance have been reported in different cattle breeds previously. For example, Namchi cattle breed has genomic regions that confer resistance to trypanosome infection [27], whereas autochthonous breeds of Cameroon have a genetic background associated with resistance to gastrointestinal nematodes and tick-borne pathogens [28]. These genomic regions and genetic changes can influence the expression of cytokines and other molecules related to the immune response, giving animals a protective immune response against different pathogens.

Analysis of the correlation between serum levels of cytokines showed that *Leishmania* spp. infection changes the relationship between these ILs. Even though these correlations were not statistically significant, and more studies should be carried out in this regard, these results suggest that regulation of immune response in *Leishmania* spp. infection is much more complex. Th1 response is activated by infected dendritic cells, which produce IL-12. This activation promotes the IFN-*γ* production by Th1 cells, which increases the NO levels and kills the parasite [29]. However, in recent years, the function of Th17 cells has been reported as having a relevant role in the regulation of proinflammatory and anti-inflammatory cytokines and can modulate the host's genetic background. In fact, one key function of IL-17, produced by Th17 cells, is recruiting neutrophils to the site of infection. Therefore, the involvement of Th17 cells and IL-17 in neutrophil recruitment suggests that Th17 cells may play a significant role in the immune response against *Leishmania* spp. infections [30]. The pathway of Th17 cells seems to be to activation of iNOS and IL-1*β*, IL-6, and TNF-*α* production [31]. However, the interaction between Th17 and Th1 pathways and the cytokines produced is still unknown. Finally, statistically significant positive correlation was found between IL-1*β* and IL-6 in Modicana cows, suggesting high activation of Th17 response in these animals.

## 5. Conclusions

For the first time, seroprevalence of *Leishmania* spp. infection was evaluated in cattle of Mediterranean region. The results showed moderate seroprevalence (17.33%), which did not differ among the bovine breeds studied. A study of cytokine serum levels indicated relevant differences between the breeds included, suggesting different immune responses. Specifically, an autochthonous Sicilian breed, Modicana, had high levels of IL-1*β*, one of the most relevant cytokines involved in the control of this and other infections. Given that studies in this regard are scarce, future studies are necessary to analyze the immune response capacity of this breed against this and other infections, to determine its possible resistance to diseases.

## Figures and Tables

**Table 1:** Seroprevalence of *Leishmania* spp. according to the bovine breed.

Bovine breed	Number of infected animals (%)	*p*-Value
Holstein	2 (11.76%)	0.7882e
Simmental	7 (18.92%)	
Modicana	4 (19.05%)	
Overall	13 (17.33%)	

**Table 2:** Cytokine serum levels (mean ± SD) in seropositive animals according to the bovine breed.

Cytokine serum levels (mean ± SD) (pg/mL)	Bovine breed			*p*-Value
	Holstein	Simmental	Modicana	
IL-1*β*	24.37 ± 4.41^a^	24.04 ± 7.00^a^	33.02 ± 11.75^b^	**<0.05**
IL-6	6.50 ± 0.04	14.51 ± 19.21	8.50 ± 2.47	0.723
IL-10	729.18 ± 514.16	2821.57 ± 5437.43	765.29 ± 1501.39	0.697
IFN-*γ*	22.00 ± 1.38	22.00 ± 3.22	23.90 ± 3.89	0.646
TNF-*α*	8.33 ± 0.33	10.97 ± 4.24	11.83 ± 8.70	0.785

*Note:* Different superscript in the same row means statistical differences. The bold value is statistically significant according to the statiscal analysis realized.

Abbreviations: IFN, interferon; IL, interleukin; TNF, tumor necrosis factor.

**Table 3:** Cytokine serum levels (mean ± SD) in seronegative animals according to the bovine breed.

Cytokine serum levels (mean ± SD) (pg/mL)	Bovine breed			*p*-Value
	Holstein	Simmental	Modicana	
IL-1*β*	39.13 ± 20.65	58.34 ± 95.05	76.50 ± 78.02	0.413
IL-6	12.98 ± 9.54^a^	6.94 ± 1.72^b^	10.51 ± 6.63^a,b^	**<0.05**
IL-10	760.30 ± 2417.08	8193.480 ± 29,869.11	307.02 ± 1192.94	0.361
IFN-*γ*	60.50 ± 150.25	23.60 ± 6.50	23.58 ± 11.99	0.248
TNF-*α*	13.02 ± 8.89	9.82 ± 3.57	10.69 ± 3.76	0.177

*Note:* Different superscript in the same row means statistical differences. The bold value is statistically significant according to the statiscal analysis realized.

Abbreviations: IFN, interferon; IL, interleukin; TNF, tumor necrosis factor.

**Table 4:** Matrix correlation between serum levels (pg/mL) of five cytokines analyzed in seropositive animals.

Cytokine	IL-1*β*	IL-6	IL-10	IFN-*γ*	TNF-*α*
IL-1*β*	1	**−0.23**	−0.19	**0.25**	**−0.05**
IL-6	—	1	−0.20	0.45	**−0.14**
IL-10	—	1	1	−0.30	0.11
IFN-*γ*	—	—	—	1	**−0.44**
TNF-*α*	—	—	—	—	1

*Note:* This table shows the Pearson correlation coefficients. Coefficients that sign change between seropositive and seronegative animals are shown in bold. No statistical significance was found in these correlations.

Abbreviations: IFN, interferon; IL, interleukin; TNF, tumor necrosis factor.

**Table 5:** Matrix correlation between serum levels (pg/mL) of five cytokines analyzed in seronegative animals.

Cytokine	IL-1*β*	IL-6	IL-10	IFN-*γ*	TNF-*α*
IL-1*β*	1	**0.15**	−0.09	**−0.04**	**0.17**
IL-6	—	1	−0.04	0.22	**0.13**
IL-10	—	—	1	−0.03	0.04
IFN-*γ*	—	—	—	1	**0.13**
TNF-*α*	—	—	—	—	1

*Note:* This table shows the Pearson correlation coefficients. Coefficients that sign change between seropositive and seronegative animals are shown in bold. No statistical significance was found in these correlations.

Abbreviations: IFN, interferon; IL, interleukin; TNF, tumor necrosis factor.

## Data Availability

The data that support the findings of this study are available from the corresponding author upon reasonable request.

## Supporting Information

Supporting Information Table S1. This file shows the raw data of all animals included in this study and the data obtained for serum levels of cytokines included.

## References

[1] Akhoundi M., Downing T., Votýpka J. (2017). *Leishmania* Infections: Molecular Targets and Diagnosis. *Molecular Aspects of Medicine*.

[2] Pace D. (2014). Leishmaniasis. *Journal of Infection*.

[3] Mann S., Frasca K., Scherrer S. (2021). A Review of Leishmaniasis: Current Knowledge and Future Directions. *Current Tropical Medicine Reports*.

[4] Maia C., Dantas-Torres F., Campino L. (2018). Parasite Biology: the Reservoir Hosts. *The Leishmaniases: Old Neglected Tropical Diseases*.

[5] Montaner-Angoiti E., Llobat L. (2023). Is Leishmaniasis the New Emerging Zoonosis in the World?. *Veterinary Research Communications*.

[6] Vioti G., Leonel J. A. F., Lemes K. M. (2019). Molecular Detection of *Leishmania* spp. in Cattle From Brazil by Means of PCR Using Internal Transcribed Spacer 1. *Revista Brasileira de Parasitologia Veterinária*.

[7] Rezaei Z., Pourabbas B., Asaei S. (2022). Livestock Infected With *Leishmania* spp. in Southern Iran. *Parasites & Vectors*.

[8] Singh N., Mishra J., Singh R., Singh S. (2013). Animal Reservoirs of Visceral Leishmaniasis in India. *Journal of Parasitology*.

[9] Bhattarai N. R., Van der Auwera G., Rijal S. (2010). Domestic Animals and Epidemiology of Visceral Leishmaniasis, Nepal. *Emerging Infectious Diseases*.

[10] Alam M. Z., Rahman M. M., Akter S., Talukder M. H., Dey A. R. (2018). An Investigation About the Possible Role of Cattle and Goats as Reservoir Hosts for *Leishmania donovani* in Bangladesh. *Journal of Vector Borne Diseases*.

[11] Rohousova I., Talmi-Frank D., Kostalova T. (2015). Exposure to *Leishmania* spp. and Sand Flies in Domestic Animals in Northwestern Ethiopia. *Parasites & Vectors*.

[12] Mukhtar M. M., Sharief A. H., el Saffi S. H. (2000). Detection of Antibodies to *Leishmania donovani* in Animals in a Kala-Azar Endemic Region in Eastern Sudan: A Preliminary Report. *Transactions of the Royal Society of Tropical Medicine and Hygiene*.

[13] Junsiri W., Wongnarkpet S., Chimnoi W. (2017). Seroprevalence of *Leishmania* Infection in Domestic Animals in Songkhla and Satun Provinces, Southern Thailand. *Tropical Biomedicine*.

[14] Remadi L., Chargui N., Jiménez M. (2020). Molecular Detection and Identification of, *Leishmania*, DNA and Blood Meal Analysis in *Phlebotomus* (Larroussius) Species. *PLOS Neglected Tropical Diseases*.

[15] Abbate J. M., Maia C., Pereira A. (2020). Identification of Trypanosomatids and Blood Feeding Preferences of Phlebotomine Sand Fly Species Common in Sicily, Southern Italy. *PLOS ONE*.

[16] Calzolari M., Romeo G., Bergamini F., Dottori M., Rugna G., Carra E. (2022). Host Preference and *Leishmania Infantum* Natural Infection of the Sand Fly *Phlebotomus perfiliewi* in Northern Italy. *Acta Tropica*.

[17] Tolentino N., Pinheiro G. R. G., Ottino J. (2019). Serological Evidence of *Leishmania* Infection by Employing ELISA and Rapid Tests in Captive Felids and Canids in Brazil. *Veterinary Parasitology: Regional Studies and Reports*.

[18] Díaz-Sáez V., Merino-Espinosa G., Morales-Yuste M. (2014). High Rates of *Leishmania Infantum* and *Trypanosoma nabiasi* Infection in Wild Rabbits (*Oryctolagus cuniculus*) in Sympatric and Syntrophic Conditions in an Endemic Canine Leishmaniasis Area: Epidemiological Consequences. *Veterinary Parasitology*.

[19] Jusi M. M. G., Starke-Buzetti W. A., de S. Oliveira T. M. F., da S. Tenório M., de O. de Sousa L., Machado R. Z. (2011). Molecular and Serological Detection of Leishmania spp. in Captive Wild Animals From Ilha Solteira, SP, Brazil. *Revista Brasileira de Parasitologia Veterinária*.

[20] Lobsiger L., Müller N., Schweizer T. (2010). An Autochthonous Case of Cutaneous Bovine Leishmaniasis in Switzerland. *Veterinary Parasitology*.

[21] Rossi M., Fasel N. (2018). How to Master the Host Immune System? *Leishmania* Parasites Have the Solutions!. *International Immunology*.

[22] Sacks D., Noben-Trauth N. (2002). The Immunology of Susceptibility and Resistance to *Leishmania major* in Mice. *Nature Reviews Immunology*.

[23] Mougneau E., Bihl F., Glaichenhaus N. (2011). Cell Biology and Immunology of *Leishmania*. *Immunological Reviews*.

[24] Barbiéri C. L. (2006). Immunology of Canine Leishmaniasis. *Parasite Immunology*.

[25] Hosein S., Rodríguez-Cortés A., Blake D. P., Allenspach K., Alberola J., Solano-Gallego L. (2015). Transcription of Toll-Like Receptors 2, 3, 4 and 9, FoxP3 and Th17 Cytokines in a Susceptible Experimental Model of Canine, *Leishmania Infantum*, Infection. *PLOS ONE*.

[26] Curone G., Filipe J., Cremonesi P. (2018). What We Have Lost: Mastitis Resistance in Holstein Friesians and in a Local Cattle Breed. *Research in Veterinary Science*.

[27] Paguem A., Abanda B., Achukwi M. D. (2020). Whole Genome Characterization of Autochthonous *Bos taurus brachyceros* and Introduced *Bos indicus indicus* Cattle Breeds in Cameroon Regarding Their Adaptive Phenotypic Traits and Pathogen Resistance. *BMC Genetics*.

[28] Abanda B., Schmid M., Paguem A. (2021). Genetic Analyses and Genome-Wide Association Studies on Pathogen Resistance of *Bos taurus* and *Bos indicus* Cattle Breeds in Cameroon. *Genes*.

[29] Birnbaum R., Craft N. (2011). Innate Immunity and, *Leishmania*, Vaccination Strategies. *Dermatologic Clinics*.

[30] Gonçalves-de-Albuquerque S. D. C., Pessoa-E-Silva R., Trajano-Silva L. A. M. (2017). The Equivocal Role of Th17 Cells and Neutrophils on Immunopathogenesis of Leishmaniasis. *Frontiers in Immunology*.

[31] Zúñiga L. A., Jain R., Haines C., Cua D. J. (2013). Th17 Cell Development: From the Cradle to the Grave. *Immunological Reviews*.

