# Supplementary material for: Seroprevalence of Leishmania spp. in Cattle Breeds of the Mediterranean Region: Effect of the Breed in the Immune Response
Source: Transbound Emerg Dis. 2025 Mar 5;2025:3277232. doi: 10.1155/tbed/3277232 (PMC12017099; doi:10.1155/tbed/3277232)
Supplement: Supporting Information — Table S1. This file shows the raw data of all animals included in this study and the data obtained for serum levels of cytokines included. [file 3277232.f1.pdf]

sample	Leishmania	Breed	[TNF] (pg/mL)	[IL6] (pg/mL)
143	Negative	HOLST	8,384679856	8,116400095
144	Negative	HOLST	7,910498236	11,64836738
145	Negative	HOLST	27,79495055	15,75271262
146	Negative	HOLST	7,809635071	12,3211765
147	Negative	HOLST	10,13379184	10,56024888
148	Negative	HOLST	7,910498236	7,23484942
149	Negative	HOLST	25,00956944	8,84307118
150	Negative	HOLST	7,485830784	8,35463568
152	Negative	HOLST	9,267416704	7,81595728
154	Negative	HOLST	8,2504471	17,66610192
155	Negative	HOLST	15,71467924	19,86235938
156	Positive	HOLST	8,569385776	6,530698095
157	Positive	HOLST	8,098496959	6,471502295
159	Negative	HOLST	7,522390876	8,56201182
160	Negative	HOLST	35,0812975	6,68011192
161	Negative	HOLST	9,618765975	44,18000938
162	Negative	HOLST	7,413938224	7,078110495
163	Negative	MOD	18,89947835	10,71770768
164	Positive	MOD	7,504059679	8,949811375
166	Positive	MOD	7,467704191	5,355953895
167	Negative	MOD	9,563586919	11,52453528
168	Negative	MOD	8,883497119	10,17229918
169	Negative	MOD	7,890120999	9,79248848
170	Negative	MOD	7,672723324	10,6388155
171	Negative	MOD	8,119897216	7,425622
172	Negative	MOD	8,592934375	13,77464912
174	Positive	MOD	7,467704191	11,36056528
175	Negative	MOD	7,8296031	7,1092955
176	Negative	MOD	10,49555678	6,770736895
177	Negative	MOD	12,3421375	7,425622
178	Negative	MOD	19,42089843	34,86742106
179	Positive	MOD	24,88462042	8,320357855
181	Negative	MOD	13,55891612	7,457702295
182	Negative	MOD	12,48850592	7,522107055
183	Negative	MOD	7,951559616	9,384097255
184	Negative	MOD	12,37857615	6,412632055
185	Negative	MOD	7,540824375	6,38331902
186	Negative	MOD	8,077199004	11,23844238
H100	Positive	SIM	19,10681876	7,1092955
H103	Negative	SIM	11,00082567	6,710238855
H104	Positive	SIM	7,413938224	6,954184375
H105	Negative	SIM	7,770005919	13,32484302
H107	Positive	SIM	7,6345264	12,44964782
H108	Positive	SIM	11,8427381	5,568645655
H109	Negative	SIM	8,453177311	5,731582855
H111	Negative	SIM	12,89945898	5,622632495
H113	Positive	SIM	8,616585276	57,74995582

H114	Negative	SIM	8,908375644	6,650066375
H116	Negative	SIM	10,13379184	6,710238855
H117	Negative	SIM	15,66985182	8,666798655
H118	Negative	SIM	12,74858927	8,84307118
H120	Negative	SIM	8,056003351	6,92340632
H123	Negative	SIM	8,883497119	6,5010595
H128	Negative	SIM	24,7600806	5,81415022
H129	Negative	SIM	8,228433031	5,43510288
H130	Negative	SIM	7,869846064	6,892709655
H131	Negative	SIM	7,540824375	5,841835455
H66	Negative	SIM	7,378605756	6,237974695
H69	Negative	SIM	14,12419802	3,90546448
H70	Positive	SIM	8,688151791	6,066247375
H71	Negative	SIM	7,378605756	7,586837375
H73	Negative	SIM	7,849673431	6,56041808
H74	Negative	SIM	7,809635071	7,55443152
H75	Negative	SIM	9,034302799	6,831560495
H77	Negative	SIM	8,688151791	6,180406695
H79	Negative	SIM	8,9584396	6,5010595
H82	Positive	SIM	13,4797984	5,676944895
H83	Negative	SIM	8,736374311	6,295868255
H84	Negative	SIM	10,25274332	5,27753742
H85	Negative	SIM	8,592934375	8,772318
H89	Negative	SIM	12,52535378	5,75902392
H94	Negative	SIM	7,730785975	9,310902375
H95	Negative	SIM	7,431757911	6,009656055
H96	Negative	SIM	7,691975239	8,98555422
H97	Negative	SIM	7,522390876	6,62010222

[IL1b] (pg/mL)	[IL10] (pg/mL)	[IFNg] (pg/mL)
20,89119239	120,3397927	18,8015012
20,58769118	13,87038164	19,6644375
38,03003139	176,2159536	27,9555472
23,64321811	132,3771929	20,1326673
60,30210957	79,4465513	21,6811501
21,42188727	999,3716234	22,1610411
43,10452218	10,74301128	21,1778992
36,20654563	13,31339378	21,3138011
26,77255445	98,49011513	22,099379
37,67165479	9452,181753	20,6154801
30,77102622	69,28711781	603,568995
21,25191191	1092,746436	22,9722139
27,48604731	365,6126508	21,0187669
23,99407758	185,3820373	20,3103719
38,31937483	0,7432619	20,9408165
78,98275639	12,77096685	25,7689838
86,28141075	39,97125317	21,3138011
99,54941559	26,17764527	69,7633525
47,9115717	8,932601853	28,8972471
50,95985255	3017,295414	24,7393695
20,35290983	77,68910606	21,6233183
20,35198253	0,688340982	20,3103719
178,6866173	2,125710862	20,8639435
23,64321811	61,50951398	25,5992184
23,61220251	5,124785084	22,0381957
20,3585625	4935,415101	20,9150725
32,31786413	32,10780653	22,1301502
20,50486295	50,30389508	20,4253273
26,13778263	6,25073018	19,6267142
139,24868	1,77729311	19,7219201
228,1859079	3,619564628	19,8201184
40,9513113	2,817118158	19,8201184
51,05472006	17,85904488	19,8201184
143,204454	21,76018984	20,8132933
20,40929683	6,25073018	20,4020968
20,36371554	0,112577393	19,292222
29,64191021	2,217998414	20,1326673
235,1597615	0,484568268	19,7219201
20,40929683	3,261532	20,6154801
20,72300535	-0,200647417	22,1301502
39,80132063	230,5602152	19,645516
210,3089793	174,7181985	27,1032685
22,77660205	31,62324163	20,5432751
20,96139147	4935,415101	20,004416
36,41188371	1,94740371	19,4452794
39,20149658	17,20270999	19,7608403
21,06731723	4,131141982	27,3830524

33,1008023	83,95491613	20,9666803
20,37417261	1701,328212	28,3962293
23,10739559	118772,9631	22,6715737
27,67029197	8,932601853	20,1763753
24,96731955	4,402021958	22,3489
20,40363002	34,60379727	23,9695403
175,255822	31,62324163	31,5818769
20,78835618	4077,968216	23,5665421
20,66293883	3,99951098	19,0882552
21,2357217	1,314212023	20,3331236
23,19339666	30,66855224	20,4957369
441,2640218	68,4816579	20,6887623
21,18803178	99,51435065	25,8975647
278,5928849	141,2623799	19,4104288
23,52003643	13,31339378	19,6267142
20,36788483	957,0621906	19,6267142
20,37523965	6,597886306	19,6080321
21,06731723	47,10167493	23,8951907
29,74665613	8,720982146	19,78048
22,05110395	14446,4686	19,9008315
21,14166293	117281,1398	23,8951907
20,68237386	25,33462185	20,1544614
31,44529899	5,751451673	28,3467859
25,32	-0,5201	18,409
35,73256093	7,906084149	23,9323057
20,51860155	2079,883866	20,2877399
20,81131407	109,0521359	47,2821879
22,16464434	107,963678	41,1335777
